# Machine Learning Driven by Magnetic Resonance Imaging for the Classification of Alzheimer Disease Progression: Systematic Review and Meta-Analysis

**DOI:** 10.2196/59370

**Published:** 2024-12-23

**Authors:** Gopi Battineni, Nalini Chintalapudi, Francesco Amenta

**Affiliations:** 1 Clinical Research, Telemedicine and Telepharmacy Centre School of Medicinal and Health Products Sciences University Camerino Camerino Italy; 2 Centre for Global Health Research Saveetha University Saveetha Institute of Medical and Technical Sciences Chennai India

**Keywords:** Alzheimer disease, ML-based diagnosis, machine learning, prevalence, cognitive impairment, classification, biomarkers, imaging modalities, MRI, magnetic resonance imaging, systematic review, meta-analysis

## Abstract

**Background:**

To diagnose Alzheimer disease (AD), individuals are classified according to the severity of their cognitive impairment. There are currently no specific causes or conditions for this disease.

**Objective:**

The purpose of this systematic review and meta-analysis was to assess AD prevalence across different stages using machine learning (ML) approaches comprehensively.

**Methods:**

The selection of papers was conducted in 3 phases, as per PRISMA (Preferred Reporting Items for Systematic Reviews and Meta-Analysis) 2020 guidelines: identification, screening, and final inclusion. The final analysis included 24 papers that met the criteria. The selection of ML approaches for AD diagnosis was rigorously based on their relevance to the investigation. The prevalence of patients with AD at 2, 3, 4, and 6 stages was illustrated through the use of forest plots.

**Results:**

The prevalence rate for both cognitively normal (CN) and AD across 6 studies was 49.28% (95% CI 46.12%-52.45%; *P*=.32). The prevalence estimate for the 3 stages of cognitive impairment (CN, mild cognitive impairment, and AD) is 29.75% (95% CI 25.11%-34.84%, *P*<.001). Among 5 studies with 14,839 participants, the analysis of 4 stages (nondemented, moderately demented, mildly demented, and AD) found an overall prevalence of 13.13% (95% CI 3.75%-36.66%; *P*<.001). In addition, 4 studies involving 3819 participants estimated the prevalence of 6 stages (CN, significant memory concern, early mild cognitive impairment, mild cognitive impairment, late mild cognitive impairment, and AD), yielding a prevalence of 23.75% (95% CI 12.22%-41.12%; *P*<.001).

**Conclusions:**

The significant heterogeneity observed across studies reveals that demographic and setting characteristics are responsible for the impact on AD prevalence estimates. This study shows how ML approaches can be used to describe AD prevalence across different stages, which provides valuable insights for future research.

## Introduction

The progression of Alzheimer disease (AD) affects memory, thinking, and behavioral functions over time [1]. Not only the individuals affected by the condition but also their families and caregivers, who have to cope with it daily. AD has become a major health concern worldwide because of the aging population in the last 3 decades [2,3]. The majority of cases of AD occur among older individuals, and increasing evidence suggests that a combination of genetic, lifestyle, and environmental factors is behind it [3,4]. The progression of the disease causes a slow deterioration of memory and cognitive abilities.

AD is represented by different stages of progression such as cognitively normal (CN) [5], significant memory concern (SMC) [6], early mild cognitive impairment (EMCI) [7], mild cognitive impairment (MCI) [8], and late mild cognitive impairment (LMCI) [7,8]. Biomarkers could help detect individuals at risk of AD before symptoms occur. Cerebrospinal fluid (CSF) testing is considered the most reliable marker of progression of AD. Brain neuroimaging like computerized tomography (CT), magnetic resonance imaging (MRI), and positron emission tomography (PET), blood tests, and genetic testing are attracting increasing attention as important markers of this pathology [1,9,10]. CSF biomarkers such as β-amyloid 42 and tau and phosphor tau are key indicators of AD [11]. An MRI or CT scan can reveal structural changes associated with AD, while a PET scan can reveal amyloid plaques and tau tangles in the brain [12]. The early diagnosis of AD can be aided by the identification of novel biomarkers, the identification of hidden data patterns, and the generation of hypotheses [13-16]. Machine learning (ML)–based predictive models can help us detect early signs of AD, improve diagnostic accuracy, and enable timely interventions [16,17].

ML applications in medicine have received significant attention for their potential in disease detection and diagnosis [18]. ML models have been proposed in existing literature to improve diagnostic accuracy for early detection of AD [19-21]. It is said that ML algorithms aid in forecasting outcomes for patients with AD, diagnosing illnesses, and tailoring treatments [15]. ML models have been reported to be able to predict patient readmissions, which allows health care providers to allocate resources more efficiently and improve patient outcomes [15,22]. In addition, deep learning (DL) algorithms can examine medical images, like CT scans or MRIs, to aid in identifying abnormalities [23-25]. The application of DL techniques to conventional MRI could reduce patient burden, risk, and cost when extracting biomarker information [26,27].

DL-based neural networks contribute significantly to AD detection [28,29]. Hierarchical representations can be learned by neural networks and achieve promising results in AD, especially when applied to neuroimaging data [30,31]. Their role includes assisting in the discovery of new AD biomarkers and analyzing large datasets to identify patterns and correlations that are indicative of AD progression [32]. Convolutional neural networks (CNNs) are used in the analysis of AD image data in the form of MRI [33], PET [34], and CT scans [35]. CNNs can automatically extract relevant features from complex imaging data and learn hierarchical representations of subtle AD patterns.

Advanced techniques like Gradient-Weighted Class Activation Mapping after CNN model training highlight important regions of the input MRI brain image [36,37]. The brain areas in these regions are responsible for influencing the model’s AD prediction. These techniques bridge the gap between accuracy and interpretability in AD detection. Moreover, recurrent neural networks are capable of analyzing temporal data, such as longitudinal studies examining cognitive decline over time [38]. Predicting cognitive decline trajectories and future outcomes is possible through the capture of sequential dependencies in data [38,39]. Multimodal data integration can enhance the accuracy of AD detection models, resulting in a more comprehensive view of the patient’s condition [40].

The role of ML models in the early diagnosis of AD has not been determined through extensive review of ML algorithms and meta-analysis. The accuracy and efficiency of AD diagnosis can be enhanced by using advanced algorithms and models as well as careful feature selection and extraction. However, the level of reliability of these techniques is a significant factor. The objective of this study is to address the knowledge gap by conducting a systematic review and meta-analysis of ML applications for AD detection, which aim to establish their role in improving diagnostic accuracy and patient outcomes. The main contribution of this study is (1) assessing the role of image feature selection methods in achieving competitive accuracy in AD classification modeling, (2) examining the ML methods that can be used to detect AD with the help of magnetic resonance image modeling, and (3) identifying the best ML classifier based on accuracy metrics.

## Methods

This study was conducted by identifying, selecting, and analyzing relevant studies, which included a literature search, screening document inclusion criteria, and tools for risk bias assessment.

### Search Strategy

A systematic search was carried out using libraries such as PubMed (MEDLINE), Scopus, and Web of Science. The search followed the PRISMA (Preferred Reporting Items for Systematic Reviews and Meta-Analysis) 2020 guidelines to maintain transparency, authenticity, and completeness of details of reporting [41]. The PRISMA checklist of this paper can be found in Multimedia Appendix 1. This search was carried out over the last 15 years and was centered on published studies specific to early-stage AD detection and classification (between January 2010 and March 2024). Limiting our review to the last 15 years of publication allowed us to focus on papers reflective of current trends in research.

The search strategy used the following keywords: “Alzheimer’s disease,” “machine learning,” “early detection,” “diagnostic accuracy,” “diagnosis,” “predictive models,” “biomarkers,” “deep learning,” “diagnostic accuracy,” “feature selection,” “AD biomarkers,” and “ML models.” The search strategy was (“machine learning” OR “artificial intelligence” OR “classification”) AND “Alzheimer’s disease” AND “MRI” AND “diagnosis” AND “classification.”

### Inclusion and Exclusion Criteria

Full-text papers in the English language were considered. We have included in this study only published papers in peer-reviewed journals. The majority of the papers analyzed were centered on MRI data combined with ML models in AD diagnosis. Selected studies included patients diagnosed with early-stage AD and healthy controls. The papers published with a title or abstract containing at least 1 abovementioned keyword were considered for inclusion.

Papers written in a language other than English were excluded. We excluded studies that were not specifically conducted in the context of AD diagnosis using MRI and were not primarily focused on ML models. Papers published before 2010 were not considered. Studies in which ML in MRI was not explicitly linked to clinical diagnosis, medical training, or initiatives to improve AD diagnosis were excluded. This review excluded studies using PET and CT scans because the primary focus was on ML in MRI, which is specifically linked to clinical diagnosis, medical training, and initiatives to enhance AD diagnosis. The selection process excluded review papers, conference proceedings, and gray literature reports.

### Paper Screening

Multiple stages were involved in the paper selection process. The results of the systematic search were documented in a spreadsheet using the above strategy. The selected papers were equally distributed among the authors, and each paper was screened by examining titles and abstracts to identify potentially relevant publications. The selected papers were then reviewed comprehensively according to predefined inclusion and exclusion criteria in the subsequent phase. To facilitate synthesis, relevant information was extracted and organized in a tabular format, covering study design, datasets, performance metrics, model validation, and feature selection. As a result, a summary of each study’s main findings to discern trends, patterns, and common themes was done.

### Quality and Publication Bias

The Newcastle-Ottawa Scale [42] was used to assess the study quality based on different factors such as selection, comparability, and outcome, providing a structured approach to gauge the risk of bias. In terms of quality, scores ranged from very poor (0-3) to moderate (4-6) to excellent (7-9). The papers meeting the score (Newcastle-Ottawa Scale≥7) were only considered for final review. Two authors (GB and NC) independently assessed the quality, and any discrepancies were resolved through discussion or consultation with a third author (FA).

### Statistical Analysis

The statistical tests Egger regression [43] and Begg rank correlation [44] were used to address the potential bias of publications. To assess the strength of our findings against potential biases or variations in study characteristics, sensitivity analyses were performed. Lower methodological quality or different study designs were excluded. To identify the effect size measures and quantify the strength or magnitude of the relationship between variables or the magnitude of differences between AD groups, we applied the “PLOGIT” function to the logit transformation of the proportion [45]. The logit transformation is commonly used when dealing with proportions or probabilities, especially when they are bounded between 0 and 1. An inverse variance method has been applied that specifies the method for pooling effect sizes. There were 2 types of models considered in the meta-analysis: fixed effects and random effects. Using the fixed effects model when we observed a low level of heterogeneity, the test is not statistically significant.

The random effects model (REM) was considered for the heterogeneity test with statistical significance [46]. By calculating *T*^2^, the amount of heterogeneity between the true effect sizes of different studies was quantified. An estimation method using a restricted maximum likelihood estimator that maximizes the likelihood function while accounting for other parameters of the model was used [47]. *I*^2^ and Cochran *Q* statistic tests were conducted to assess the heterogeneity among the effect sizes of individual studies [48,49]. The measures of heterogeneity (*T*^2^ and *I*^2^) indicate the variability in AD prevalence estimates across the studies [50].

The prevalence of patients with AD across different subgroups within the overall population was also investigated. Subgroup analysis enables the identification of factors that can influence prevalence estimates and provide insight into the sources of heterogeneity [51]. A subgroup-specific meta-analysis model was used to calculate the pooled prevalence estimates for each subgroup, followed by a comparison of the prevalence estimates across subgroups to assess whether there were any significant differences. Data were subgrouped into 4 category-based AD classifications namely, 2-group classification, 3-group classification, 4-group classification, and 6-group classification. The 2-group classification involved individuals either without dementia (nondemented, ND) or with dementia (demented, AD). The 3-group classification includes CN, MCI, and AD. The 4-group classification comprises ND, mildly demented (MD), moderately demented (MoD), and AD. Meanwhile, the 6-group classification involves CN, SMC, EMCI, MCI, LMCI, and AD. Each subgroup data was recorded separately into a Microsoft Excel spreadsheet, which was further supplied as input to R software (version 4.3.3; R Foundation for Statistical Computing). For prevalence and summary meta-analysis, we used the “meta prop” functions available in the *meta* package.

## Results

### Search Outcomes

**Figure 1:**
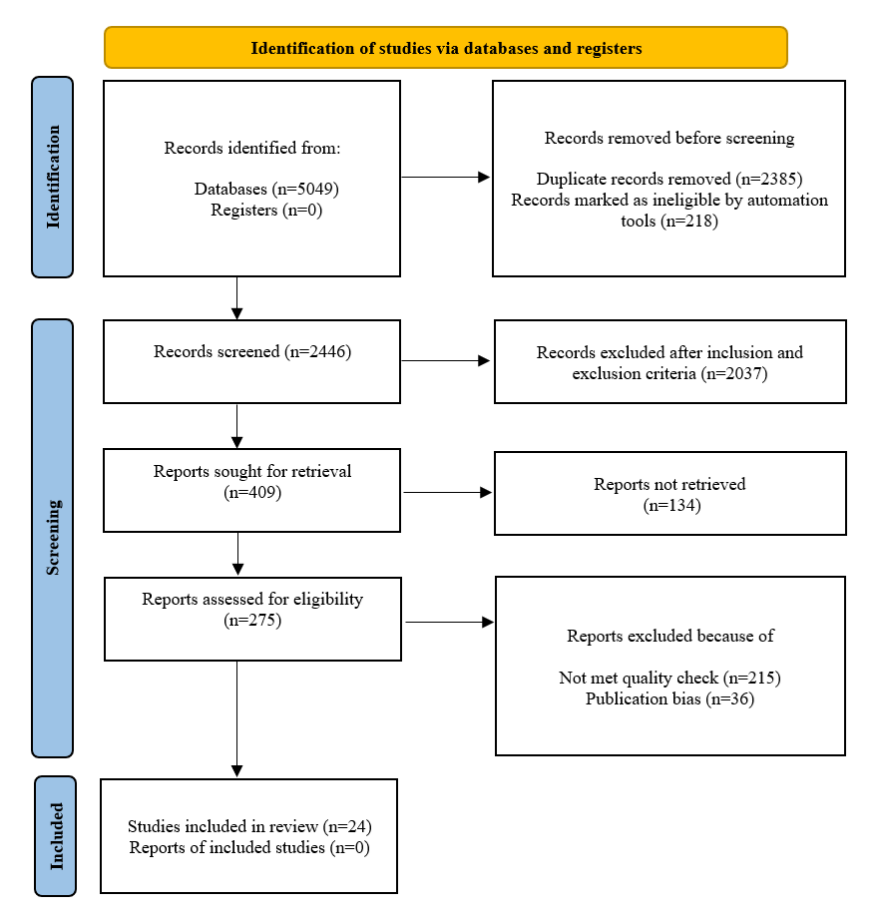
Paper screening procedure flowchart.

During the identification phase, 5049 records were obtained from 3 major scientific databases using the given search strategy. Following the removal of duplicates (n=2355) and the assessment of ineligibility using tools (n=218), 2446 records were included in the screening stage. The inclusion and exclusion criteria determined that 2037 records were ineligible. We further screened 409 records, with 134 being excluded due to lack of full-text availability. In total, 251 records from the remaining 275 were excluded due to low-quality scores and publication bias. A total of 24 papers were included in the final analysis. Details on the procedures for selecting papers are summarized in Figure 1.

### Data Sources

The data collected for this study were collected from various geographical locations and may have included memory clinics and neurology departments, suggesting a focus on cognitive impairment and related conditions. Table 1 displays the distribution of AD imaging sample data along with data sources.

**Table 1:** Participants’ data collected from different sources.

Data source	AD^a^, n/N (%)	Reference
ADNI^b^	33/204 (16.17)	[52]
Tianjin First Central Hospital, China	27/56 (48.21)	[53]
ADNI and AIBL^c^	1673/3335 (50.16)	[54]
OASIS^d^ 3	1077/3979 (27.06)	[55]
ADNI	70/210 (30)	[56]
ADNI	193/818 (23.59)	[57]
ADNI	3200/6400 (50)	[58]
ADNI	186/805 (23.10)	[59]
Kaggle	3200/6400 (50)	[60]
ADNI	231/432 (50)	[61]
Kaggle	3200/6400 (50)	[62]
Shanghai Pudong New Area People’s Hospital	55/119 (46.21)	[63]
ADNI	268/1048 (25.57)	[64]
ADNI and NACC^e^	1170/4644 (25.19)	[65]
Kaggle and ADNI	390/1310 (29.77)	[66]
ADNI	584/1421 (41.1)	[67]
ADNI	25/138 (18.11)	[68]
OASIS 1	78/150 (52)	[69]
Kaggle	3200/6400 (50)	[70]
Memory clinic of the neurology department in Nanfang Hospital	44/180 (24.44)	[71]
ADNI	118/245 (48.16)	[72]
ADNI	24/142 (16.90)	[73]
ADNI	1077/3979 (27.06)	[74]
ADNI	260/560 (46.42)	[75]

^a^AD: Alzheimer disease.

^b^ADNI: Alzheimer’s Disease Neuroimaging Initiative.

^c^AIBL: Australian Imaging Biomarkers and Lifestyle Flagship Study of Ageing.

^d^OASIS: Open Access Series of Imaging Studies.

^e^NACC: National Alzheimer’s Coordinating Center.

The analyzed studies collected image data from various sources such as Alzheimer’s Disease Neuroimaging Initiative (ADNI) [76], Open Access Series of Imaging Studies (OASIS) [77], Australian Imaging Biomarkers and Lifestyle Flagship Study of Ageing [78], and public domains like Kaggle [79]. ADNI datasets were used more often for image collection [52,54,56-59,61,64-68,72-75,80]. The purpose of ADNI is to develop biomarkers for early detection and AD tracks through a multicenter study involving clinical imaging, genetics, and biochemistry. The studies that use ADNI datasets aim to detect AD at its prime stage. One study jointly applied 2 image datasets from ADNI and Australian Imaging Biomarkers and Lifestyle Flagship Study of Ageing [54].

OASIS brains aim to make it possible for anyone to access neuroimaging datasets of the brain through an initiative known as Open Access to Neuroimaging Datasets. Through this project, researchers can access and use a variety of brain imaging data for free. This resource assists neuroscience researchers in advancing their research by providing a comprehensive collection of brain imaging datasets. Cross-sectional OASIS 1 data were used by researchers for hypothesis-driven analysis, neuroanatomical atlases, and segmentation algorithms [69]. In another study, OASIS-3 was integrated with longitudinal neuroimaging, clinical, cognitive, and biomarker data [55]. The use of public datasets or participation in Kaggle competitions related to AD research helps as a platform for data science competitions and datasets [70]. Three studies collected data from 3 hospitals in China [53,63,71]. The findings indicate that a diverse dataset from multiple sources, such as clinical settings and publicly available datasets, could provide a comprehensive basis for AD research and analysis.

### Study Characteristics

#### AD Stages

Table 2 presents a summary of various studies, which includes authors, publication year, AD stages, preprocessing techniques, classifiers, validation methods, and the best-performing model. Four studies have examined the progression of AD over 6 stages to gain a better understanding of how diseases develop and change [52,54,68,73]. Seven studies examined 4 groups of AD stages analyzing neurobiological mechanisms behind cognitive decline or exploring nonpharmacological treatments [55,58-60,64,66,70]. Similarly, 7 works associated with 3-stage classification studies involved patients with CN, MCI, and AD [56,57,62,65,67,71,74]. These studies were mainly focused on the early detection of dementia with subtle differences in biomarkers and cognitive performance. Moreover, the ML models used in the study predicted AD progress in estimating the transition from MCI to dementia. Finally, 6 studies associated a binary or 2-stage classification of AD with ML models to identify biomarkers that predict treatment response or disease progression [53,61,63,69,72,75]. This enables more effective targeted therapies and biomarker-driven clinical trials to be developed.

**Table 2:** Machine learning models and their characteristics.

	Author	Year	AD^a^ stages	Image preprocessing methods	ML^b^ models incorporated	Validation	Diagnosis accuracy (%)	Best model
1	Alorf and Khan [52]	2022	6	Normalization and smoothing	GLMICA^c^	K-fold (10)	84.03	BC-GCN^d^
2	Chen et al [53]	2017	2	Diffusivity and kurtosis mapping and ROI^e^	SVM^f^	K-fold (10)	96.23	SVM with DKI^g^
3	Mofrad et al [54]	2021	6	LME^h^ for ROI extraction	SVC^i^	K-fold (15)	69-75	SVC
4	EL-Geneedy et al [55]	2023	4	Image normalization	DenseNet121^j^, ResNet50^k^, VGG16^l^, EfficientNetB7, and InceptionV3	K-fold (10)	99.68	Customized CNN^m^ model
5	Hazarika et al [56]	2022	3	Histogram-based approach	20 Different DL^n^ models	K-fold (10)	90.22	DenseNet121
6	Khan et al [57]	2022	3	SMOTE^o^	16 Different ML models	K-fold (10)	90.24	SVM with DKI
7	Sorour et al [58]	2024	4	Image normalization and labeling	CNN, LSTM^p^, SVM, and VGG16	K-fold (10)	99.92	CNN-LSTM
8	Abdelaziz et al [59]	2021	4	Interpolation	CNN	K-fold (10)	98.22	CNN
9	Sharma et al [60]	2022	4	VGG16	Neural network with VGG16 feature extractor	K-fold (10)	90.4	VGG16
10	Nguyen et al [61]	2022	2	Augmentation	3D-ResNet, XGB^q^	K-fold (5)	96.20	XGB
11	Saleh et al [62]	2023	3	CNN feature extraction	DenseNet121, 169, and 201	K-fold (10)	96.05	DenseNet201
12	Yang et al [63]	2022	2	Recursive feature elimination	Recursive random forest (RF)	K-fold (10)	97	RF
13	El-Sappagh et al [64]	2021	4	SMOTE	SVM, KNN^r^, DT^s^, NB^t^, RF	K-fold (10)	87.76	RF
14	Liu et al [65]	2022	3	Unified segmentation	3D CNN	Holdout and external validation	85.12	3D CNN
15	Elgammal et al [66]	2022	4	Generalization	KNN	Multifractal geometry	99.4	KNN
16	Das et al [67]	2021	3	Skull stripping, intensity normalization, corpus callosum segmentation	SVM	K-fold (100)	90	SVM
17	Chelladurai et al [68]	2023	6	Gray-level co-occurrence matrix	RF, XGB, DT, SVM, MLP^u^	Evaluation metrics	99.44	MLP
18	Battineni et al [69]	2021	2	Outliers’ detection	RF, GNB^v^, LR^w^, SVM, gradient boosting, and Ada boosting	K-fold (10)	97.58	Gradient boosting
19	Sharma et al [70]	2022	4	Normalization and augmentation	SVM, XGB, GNB	Not mentioned	89.89	SVM
20	Long et al [71]	2023	3	MRMR^x^ algorithm in combination with the SFC^y^ method	SVM, ANN^z^	K-fold (10)	80.36	SVM
21	Wang et al [72]	2023	2	Deep features extraction	CNN	K-fold (5)	98.86	CNN
22	Tajammal et al [73]	2023	6	Augmentation	VGG16, ResNet18, Alex Net, Inception V1, Custom CNN	Not mentioned	96.2	Custom CNN
23	Golovanevsky et al [74]	2022	3	Unified hyperparameter tuning	Multimodal	K-fold (3)	96.88	Multimodal AD diagnosis framework
24	Li and Yang [75]	2021	2	Transfer learning	SVM, VGG Net^aa^, ResNet	K-fold (5)	95	VGG Net, ResNet

^a^AD: Alzheimer disease.

^b^ML: machine learning.

^c^GLMICA: generalized linear model incorporating covariates analysis.

^d^BC-GCN: brain connectivity–based graph convolutional network.

^e^ROI: region of interest.

^f^SVM: support vector machine.

^g^DKI: diffusion kurtosis imaging.

^h^LME: linear mixed-effects model.

^i^SVC: support vector classifier.

^j^DenseNet: dense convolutional network.

^k^ResNet: residual network.

^l^VGG: Visual Geometry Group.

^m^CNN: convolutional neural network.

^n^DL: deep learning.

^o^SMOTE: Synthetic Minority Oversampling Technique.

^p^LSTM: long short-term memory.

^q^XGB: extreme gradient boosting.

^r^KNN: k-nearest neighbor.

^s^DT: decision tree.

^t^NB: Naïve Bayes.

^u^MLP: multilayer perceptron.

^v^GNB: Gaussian Naive Bayes.

^w^LR: logistic regression.

^x^MRMR: minimum redundancy maximum relevance.

^y^SFC: sparse functional connectivity.

^z^ANN: artificial neural network.

^aa^VGG Net: Visual Geometry Group network.

#### Feature Engineering Techniques

Feature engineering plays an important contribution in brain image analysis [81]. Various feature techniques were discussed to tackle challenges in AD classification, such as class imbalance, feature extraction, robustness, and generalization. ConvNet or CNN was designed for processing grid-like data, such as images, using convolutional layers to learn spatial hierarchies of features automatically [62]. Visual Geometry Group (VGG16) uses 3×3 convolution filters to construct a 16-layer CNN architecture and is known for its simplicity and high performance in image classification tasks [60]. Models like multilayer perceptron, Dense Net, Efficient Net, and residual network in AD classification lie in their ability to effectively handle deep neural networks for feature extraction and classification, which is crucial in analyzing complex brain magnetic resonance images for AD detection. Support vector machine (SVM) is a supervised learning algorithm used for AD classification, and it constructs hyperplanes in a high-dimensional space to separate different classes. In contrast, diffusion kurtosis imaging (DKI) is an MRI procedure that captures non-Gaussian diffusion, giving insight into tissue microstructure and facilitating better brain mapping. These techniques range from basic normalization [55,58,70], outlier detection [69], interpolation [59], and transfer learning [75] to more advanced methods such as data augmentation [61,70,73], feature extraction using DL models like VGG16 [60], deep feature extraction [72], ConvNet [62], and statistical modeling for region of interest extraction [54]. Another paper extracted features related to corpus callosum atrophy for AD diagnosis [67]. A single study investigated texture analysis in brain images using the Gabor and gray-level co-occurrence matrix [52]. For feature selection and analysis of functional connectivity patterns, another investigation used the minimum redundancy maximum relevance algorithm alongside the sparse functional connectivity method [55]. Unified hyperparameter tuning was applied to optimize model parameters across algorithms and settings [58].

#### Classifiers

Supervised models like SVM were used by several studies for classification tasks due to their effectiveness in handling high-dimensional magnetic resonance image data and nonlinear relationships [53,54,58,64,67-69,71,75]. The generalized linear model incorporating covariates analysis was used by Alorf and Khan [52] to assess a model’s performance and generalization ability by ensuring that all data points are used during both training and validation, reducing overfitting risk and allowing more reliable model performance estimates. The authors demonstrated that MRI data can be fine-tuned to capture subtle differences in brain morphology associated with AD by using pretrained models [55].

Similarly, to learn discriminative patterns, other models like logistic regression (LR), decision tree, Gaussian Naive Bayes, and k-nearest neighbor (KNN) largely contribute to the MRI-based AD classification. The combination of these multimodal classifiers was adopted among 6 works to leverage AD early diagnosis [63,64,66,68-70]. Alternatively, CNN-based DL models have the capability of autonomous learning and represent complex patterns in magnetic resonance images. In this review were identified 2 studies that used dense convolutional network (DenseNet) [55,62] and Inception [55,73]. In total, 4 studies applied residual network [55,61,73,75], 5 studies used VGG [55,58,60,73,75], and 1 study the EfficientNet [55]. The multimodeling approaches (comparison of 16 and 20 classifiers) of CNN models were incorporated in 2 works [56,57]. Long short-term memory, another DL framework largely used in the context of MRI classification, can be used to analyze sequential data, such as time-series MRI scans, to detect temporal changes in brain structures characteristic of AD progression [58]. One study used a different approach, the multimodal neural networks for analyzing data from multiple sources or modalities [74]. Ensemble learning techniques like extreme gradient boosting (XGB), gradient boosting, and Ada boosting combine weak learners to create a more powerful classification. MRI data in 4 studies were successfully handled by the XGB classifier, which captured nonlinear relationships between features and predicted AD status accurately [61,68-70].

#### Validation Techniques

K-fold cross-validation is a common method used by most studies, where the dataset is divided into K subsets, and the model is trained and tested for K times. Testing was conducted on each subset, while the remaining ones served as training. This method can be used to assess model performance and generalization across different subsets of data. The K-fold has been used in most studies with varying values of K including 3 [74], 5 [61,72,75], 10 [52,53,55-60,62-64,69,71,72], 15 [54], and 100 [67], indicating that the total partitioning of data varies depending on the level of validation. It is important to take into account the differences between different methods of validation. A recent study used a holdout technique and external validation, dividing the dataset into training and testing sets and performing an additional test on completely new, from-scratch datasets [65]. A unique approach to data analysis that uses multifractal geometries has been introduced by Elgammal et al [66] and is likely to involve characterizing complex patterns in data using fractal-based techniques. The findings above show that many validation methods need to be considered. Therefore, adaptable methodologies are necessary when it comes to datasets and objectives. On the other hand, there are a few mentions of specific evaluation metrics [68]. The use of K-fold cross-validation remains common, but the inclusion of alternative methods such as holdout and multifractal geometry suggests a willingness to explore new approaches to evaluating model performance and ensuring the robustness of ML and data analysis tasks.

### Prevalence-Based Participant Pooling

**Figure 2:**
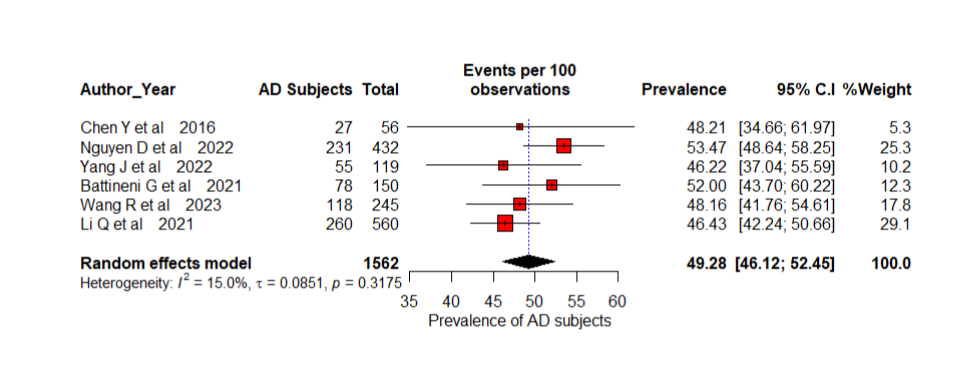
A forest plot AD diagnosis prevalence (%) among 2-stage classification using random effects model [53,61,63,69,72,75]. AD: Alzheimer disease.

**Figure 3:**
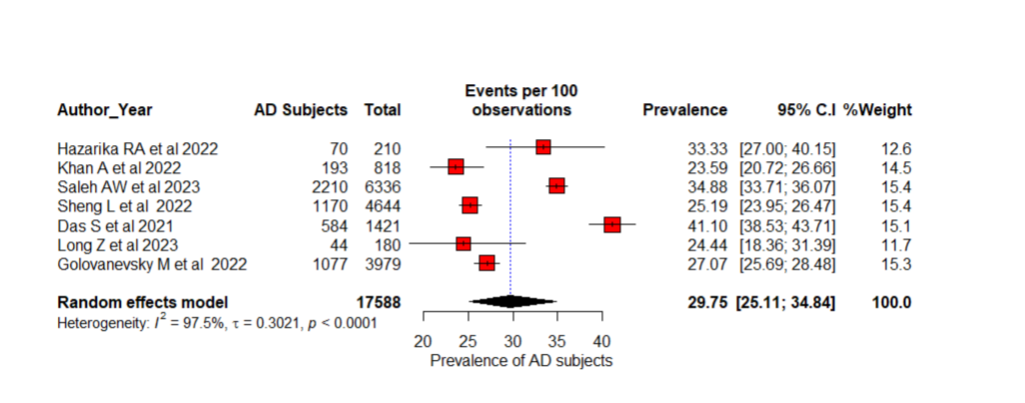
A forest plot AD diagnosis prevalence (%) among 3-stage classification using random effects model [56,57,62,65,67,71,74]. AD: Alzheimer disease.

**Figure 4:**
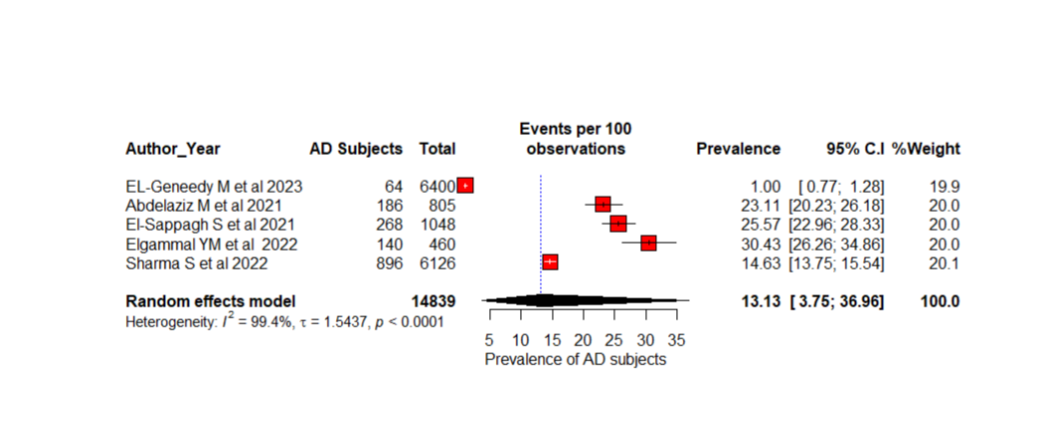
A forest plot AD diagnosis prevalence (%) among 4-stage classification using random effects model [55,59,64,66,70]. AD: Alzheimer disease.

**Figure 5:**
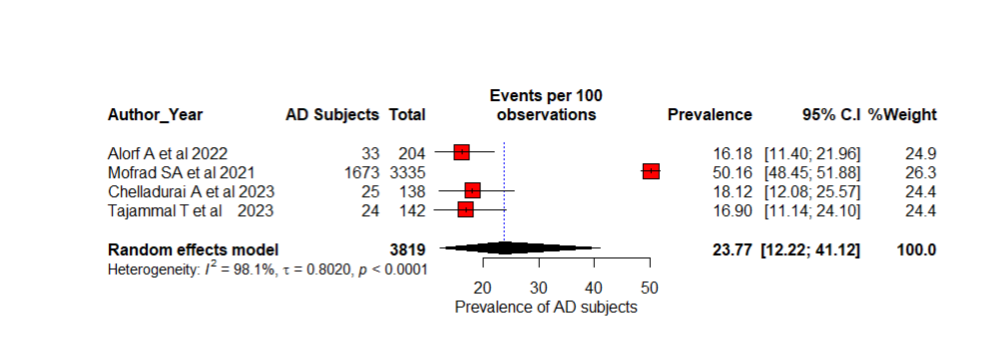
A forest plot AD diagnosis prevalence (%) among 6-stage classification using random effects model [52,54,68,73]. AD: Alzheimer disease.

There was no evidence of publication bias with Eggers (*P*=.49) or Begg (*P*=.38) tests. Figures 2-5 present the forest plot with the prevalences of participants with AD for 2, 3, 4, and 6 AD stage subgroups, respectively. Six studies with 1562 participants were identified among disease diagnoses with 2 stages including CN and AD [53,61,63,69,72,75]. The overall pooled prevalence of the REM reported 49.28% (95% CI 46.12%-52.45%; *I*^2^=15%; *P*=.32). Studies do not differ significantly in their estimates of prevalence, and the test of heterogeneity does not reveal substantial differences between them. Seven studies were identified with a total sample of 17,588 patients with AD with 3-stage AD classification including CN, MCI, and AD [56,57,62,65,67,71,73]. The overall prevalence of AD diagnosis is estimated at 29.75% (95% CI 25.11%-34.84%; *I*^2^=97%; *P*<.001). Each study provides an estimate of the AD prevalence among their respective populations with 95% CI. For example, Hazarika et al [56] found AD prevalence at 33.33% (95% CI 27%-40.15%). This indicates that if we were to combine the results of all the studies, this would be the estimated AD prevalence. *I*^2^=97% indicates that a large proportion of the total variation in prevalence estimates is due to true differences between study populations rather than random error. The significant *P* value (<.01) for the test of heterogeneity indicates that there is substantial variability in AD diagnostic prevalence estimates among the studies.

Five studies with 14,839 participants were included for the meta-analysis of 4-stage AD classifications as ND, MoD, MD, and overt AD [55,59,64,66,70]. This systematic review included 7 studies, but we excluded 2 studies [58,60] because they used the same dataset with 6400 ADNI participants. Overall prevalence estimation with REMs is 13.13% (95% CI 3.75%-36.96%; *I*^2^=99%; *P*<.001). There is significant heterogeneity in the studies based on the high *I*^2^ and significant *P* value and a considerable variation in the prevalence of AD across these studies, according to these estimates. Different research studies have found prevalence estimates ranging from 1% [55] to 30.43% [66]. The CIs indicate the degree of uncertainty in these estimates. As a result of the high degree of heterogeneity observed in the study, the true prevalence of AD may vary significantly between populations and settings. Four studies with 3819 were considered for the calculation of the overall prevalence of AD diagnosis of 6 stages such as CN, SMC, EMCI, MCI, LMCI, and AD [52,54,68,73]. The estimated prevalence for each study is ranging from 16.18% [52] to 50.16% [54]. The overall estimate of prevalence from the REM stands at 23.77% (95% CI 12.22%-41.12%; *I*^2^=0.8020; *P*<.001). One study has a substantially greater estimated proportion of AD prevalence diagnosis than the other studies [54]. Compared to others, it reported the highest prevalence of 50.16% (95% CI 48.45%-51.88%) but does not differ weights (26.3%) significantly from other studies.

Meta-analysis through forest plots provides a comprehensive way of understanding meta-analysis results. It can be argued, however, that forest plots can only display CIs by assuming a fixed significant threshold (*P*<.05). It causes a replication crisis when hypothesis tests are conducted using *P* values. Based on *P* value functions, drapery plots were proposed to resolve this problem [82]. Using a drapery plot, an average effect and a confidence curve can be identified. The x-axis shows the effect size metric, and the y-axis shows the assumed *P* value. Multimedia Appendix 2 presents the drapery plots. There is a red curve showing the overall REM, which shows the *P* values for various effect sizes. Compared to the CI of pooled effects, the shaded area represents the prediction range. The prediction range is noticeably wider than the CI for the pooled effect. It indicates that the overall pooled effect does not fully capture the variability or uncertainty across different effect sizes.

## Discussion

### Principal Findings

In this work, we conducted a systematic review and meta-analysis based on the prevalence of patients with AD among different disease progression stages. For the systematic review, 24 studies were selected, among 22 selected for the meta-analysis. Due to their association with the same dataset of ADNI and similar sample size of patients with AD, these 2 studies avoid bias in the analysis [58,60]. The studies included in this review have explored the ML applications for AD diagnosis and intended to provide an understanding of AD progression, potentially with a focus on biomarker identification.

Different preprocessing techniques used to extract relevant features including cortical thickness [83], hippocampal volume [84], and brain activity patterns [85] from magnetic resonance images associated with AD were examined. According to the research objectives and AD stages being investigated, each study applied specific image preprocessing techniques. The progression of AD has been evaluated across multiple stages in our work. An accuracy range of 69%-75% is achieved with linear mixed-effects models that account for region of interest features with interparticipant variability of hierarchical structures [54]. Using image normalization, 1 study classified AD stages with different labeling with 84.03% accuracy by ensuring consistency in intensity and spatial properties [52,86]. Combining DL models with imaging techniques like MRI and PET has shown that structural and functional changes in the brain associated with AD can be detected [87,88]. Water molecules’ diffusion properties in brain tissue can be measured using diffusivity and kurtosis mapping. The results provided insight into microstructural changes for a maximum accuracy of 96.23% [53]. By conducting magnetic resonance image normalization, the authors proposed an MRI-based DL technique for 99.68% accurate AD detection [55]. Magnetic resonance images were investigated for pixel intensity distributions to detect AD abnormalities [56].

These techniques encompass diverse methodologies ranging from normalization and smoothing to advanced mapping and feature extraction methods [89-91]. Several approaches have demonstrated high accuracy in identifying AD features, including image normalization, histogram-based approaches, and diffusion mapping [92-95]. Techniques like recursive feature elimination and outlier detection showcase promising results, emphasizing the importance of feature selection and data quality assessment in enhancing classification performance [57,63]. A similar study analyzed and segmented different tissue types within MRI scans using unified segmentation. A magnetic resonance image of the brain was segmented simultaneously into different tissue types with 85.12% accuracy [65,96]. KNN-trained data can be used to classify AD with 99.4% accuracy using the generalization method [66]. Moreover, the use of advanced DL architectures such as VGG16 [60] and ConvNet [62] for feature extraction underscores the significance of leveraging sophisticated computational tools in AD research. Augmentation methods, interpolation, and transfer learning also emerge as valuable strategies for improving classification accuracy and robustness [73-75].

By integrating statistical and ML algorithms with preprocessing techniques, AD diagnosis research further enhances its interdisciplinary nature. The CNN-long short-term memory model had an accuracy of 99.92%, followed by the multimodal AD diagnosis framework model with a precision of 96.88%. The accuracy of a customized CNN model was 99.68%, SVM with DKI was 96.23%, XGB was 96.20%, and multilayer perceptron was 99.44%. In addition, DenseNet121, CNN, DenseNet201, random forest, and gradient boosting achieved accuracy levels between 90% and 97%. While some models demonstrated higher accuracy, such as 3D CNN and SVM, others demonstrated lower accuracy, 85.12% and 80.36%, respectively.

Many ML modeling techniques have been explored, including SVM, LR, and DenseNet. Ensemble methods like gradient boosting and Ada boosting have highlighted the importance of aggregating multiple models to improve predictive accuracy and robustness, especially when dealing with complex neurological disorders like AD [69,97]. The identification of specific best-performing models further underscores the importance of optimization of methods and model selection to improve diagnostic accuracy. The use of SVM along with DKI or DenseNet201 in different studies illustrates the researchers’ tailored approach to leveraging each algorithm’s and feature representation’s strengths [98-100]. AD diagnosis is a nuanced process, where the choice of ML model can have a significant impact on model reliability and efficacy.

Data from magnetic resonance images have been analyzed using various ML models and validation techniques. To ensure robustness and generalization, the common technique used is K-fold cross-validation. Additionally, some authors have applied specific DL models along with traditional ML techniques, reflecting the diversity of approaches for modeling and validation [72,73]. Different mechanisms and approaches are used in each of these models to detect AD using magnetic resonance images. We have observed that SVM classifiers are largely used for 2-stage classification such as CN and AD [53,54,67,71]. Similarly, LR classifiers were used in other studies to assess MRI-based AD status interpretation and predictive factors for disease risk assessment. Based on learned discriminative patterns from magnetic resonance images, these models, as well as others mentioned, produce accurate AD detection predictions. Additionally, KNN can be used to identify magnetic resonance images with feature vectors similar to those associated with AD helping to detect patterns.

The meta-analysis shows that there is a great deal of variation between studies when it comes to estimating AD prevalence. The reason for this is probably because the study involved a wide range of diagnostic criteria and populations, not just prevalence rates. The prevalence estimates are diverse due to some studies focusing on specific AD stages while others cover a wider spectrum. The significant *P* values and *I*^2^ statistics show that the diagnosis of AD is highly heterogeneous and requires a nuanced understanding of its epidemiology. The challenges associated with synthesizing prevalence data from disparate sources are revealed by this analysis. The prevalence of AD is subject to complex and variable research, which leads to wider CIs in some studies. Even after trying to use REMs to account for this heterogeneity, significant variation persists, suggesting that variables like demographics, study design, and diagnostic methodology may play a significant role. The provision of more reliable estimates requires the adoption of standardized protocols and collaboration in future research efforts, which stresses the importance of rigorous methodology and careful interpretation of results.

### Comparison With Existing Reviews

There have been a few systematic reviews and meta-analyses about the importance of ML models in AD diagnosis. Table 3 summarizes the comparison between our work and the reviews that have already been published. In our analysis, we concentrated on using ML for AD diagnosis, while other studies were focused on using it for dementia forecasting [101]. In a similar study [102], the authors explored the effectiveness of both ML and DL models in AD diagnosis. In this study, the authors did not examine multistage AD cases but only the binary classification of AD. A single study [103] conducted a meta-analysis based on Wilcoxon signed rank tests and discussed multiple imaging modalities, including MRI, PET, and CSF. Despite this, there is a lack of discussion about feature selection techniques and their potential impact on ML accuracy. A prevalence-based meta-analysis on MRI-centered AD discussions is presented in our study along with an in-depth description of subcategories of AD. Our study stands out because it covers all aspects of ML in AD diagnosis, including imaging modalities and stages of AD. We reviewed and analyzed various imaging modalities, talked about feature selection methods, and delved deeper into AD subcategories in our research.

**Table 3:** Comparison of this review with existing systematic reviews.

Study	Systematic review	Meta-analysis	Imaging modalities	Feature selection	Alzheimer disease stages
[101]	✓		✓		4
[102]	✓		✓		2
[103]	✓	✓	✓		6
Our study	✓	✓	✓	✓	6

### Future Directions and Study Limitations

Data from open-access libraries such as ADNI, Kaggle, and others were used in studies, as evidenced by the analysis of datasets. Prospective validation studies should be carried out in the future to assess the accuracy of ML models for AD diagnosis across diverse populations and clinical settings. The incorporation of multimodal data, including imaging, genetics, and clinical information, into ML models can improve their accuracy and robustness in diagnosing AD and distinguishing it from other brain disorders [89]. To enhance their clinical utility and acceptability, ML models must be interpretable and explainable. It may be possible to use these models to predict the onset and AD progression based on longitudinal studies that track individuals over time [14,101]. Future research must incorporate ML models into diagnostic workflows and assess their influence on patient outcomes and health care delivery.

Despite its comprehensiveness, this study is characterized by some shortcomings. The availability and quality of data are essential for the effectiveness of ML approaches. The outcome of the meta-analysis may have been influenced by the limitations in access to complete datasets with different levels of quality. The potential for publication bias, in which studies with positive findings are more likely to be published, may lead to an overestimation of the effectiveness of ML approaches for diagnosing AD. The included studies may have experienced heterogeneity due to variations in study designs, patient populations, imaging modalities, and ML algorithms, making it difficult to draw definitive conclusions. Despite our best efforts to conduct a thorough review, some relevant studies may have been mistakenly excluded, potentially creating gaps in the analysis. The generalizability of ML models for AD diagnosis may be limited by their development and validation on specific datasets.

### Conclusions

A summary comparison of current literature on ML approaches in AD diagnosis, along with a systematic review and meta-analysis, helps to understand the prevalence of disease at different stages. Our analysis of 24 relevant papers shows a significant difference in AD prevalence estimates, as individuals progress from CN to MCI and ultimately to overt AD. We observed a pooled prevalence of 49.28% during the CN to AD transition. This was followed by 29.75% for CN, MCI, and AD, 13.13% for CN, MoD, MD, and AD, and 23.75% for CN, SMC, EMCI, MCI, LMCI, and AD. Our analysis reveals the importance of adjusting diagnostic and management strategies to minimize the impact of demographic and setting characteristics on AD prevalence estimates. Due to the heterogeneity observed across studies, it is necessary to consider various factors to accurately estimate the prevalence of AD. Our study is different from other studies by comparing it to existing systematic reviews and meta-analyses, which provide an original contribution to the topic under evaluation. Unlike previous studies that have focused on imaging modalities and AD stages, our study has comprehensively analyzed ML in AD diagnosis. Multiple imaging modalities were reviewed and analyzed, feature selection techniques were discussed, and AD subcategories were explored, focusing particularly on MRIs. Although none of the biomarkers currently available can provide a precise diagnosis of AD, using ML approaches to identify prevalence patterns across disease stages will lead to progress in AD diagnosis.

## Appendix

Multimedia Appendix 1 PRISMA (Preferred Reporting Items for Systematic Reviews and Meta-Analysis) checklist.

Multimedia Appendix 2 Drapery plots.

## Abbreviations

AD: Alzheimer disease
ADNI: Alzheimer’s Disease Neuroimaging Initiative
CN: cognitively normal
CNN: convolutional neural network
CSF: cerebrospinal fluid
CT: computerized tomography
DenseNet: dense convolutional network
DKI: diffusion kurtosis imaging
DL: deep learning
EMCI: early mild cognitive impairment
KNN: k-nearest neighbor
LMCI: late mild cognitive impairment
LR: logistic regression
MCI: mild cognitive impairment
MD: mildly demented
ML: machine learning
MoD: moderately demented
MRI: magnetic resonance imaging
ND: nondemented
OASIS: Open Access Series of Imaging Studies
PET: positron emission tomography
PRISMA: Preferred Reporting Items for Systematic Reviews and Meta-Analysis
REM: random effects model
SMC: significant memory concern
SVM: support vector machine
VGG: Visual Geometry Group
XGB: extreme gradient boosting
