# Supplementary material for: Machine Learning Driven by Magnetic Resonance Imaging for the Classification of Alzheimer Disease Progression: Systematic Review and Meta-Analysis
Source: JMIR Aging. 2024 Dec 23;7:e59370. doi: 10.2196/59370 (PMC11704653; doi:10.2196/59370)
Supplement: Multimedia Appendix 2 [file aging_v7i1e59370_app2.docx]

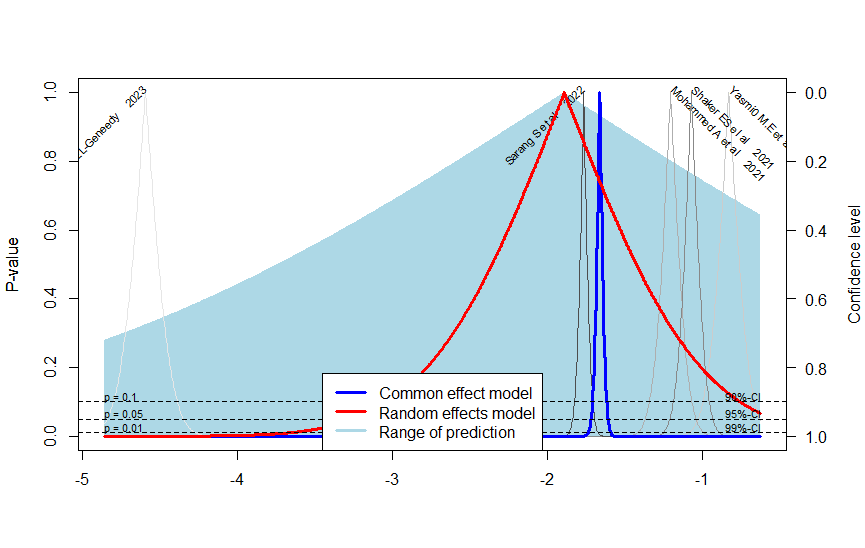
**Figure S1.** Drapery plot representation of 2-stage AD classification studies.


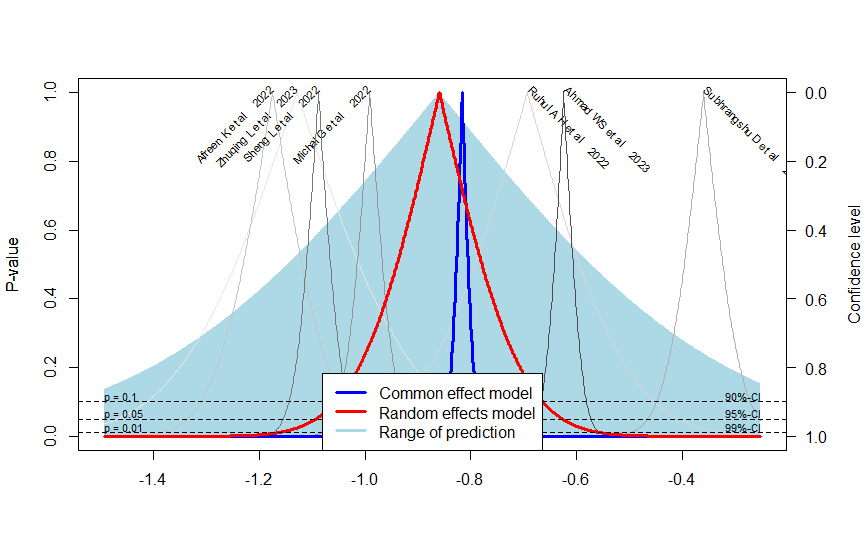


**Figure S2.** Drapery plot representation of 3-stage AD classification studies.


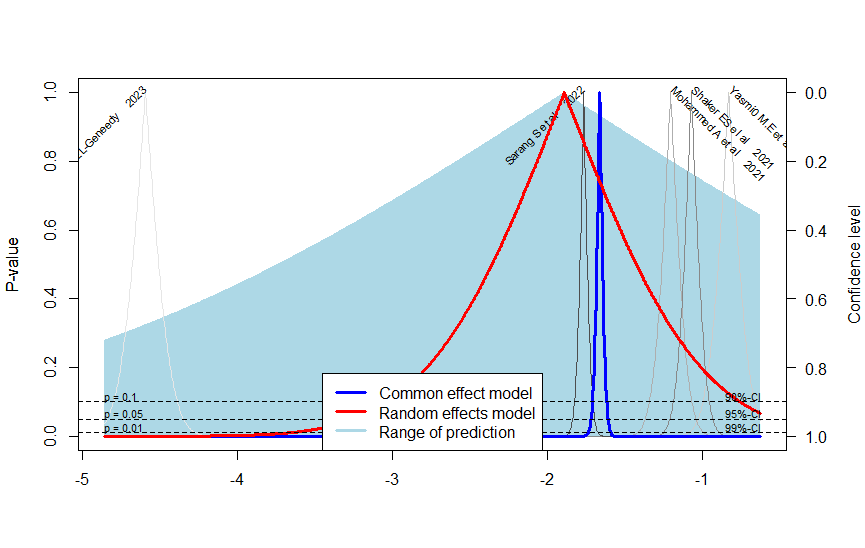


**Figure S3.** Drapery plot representation of 4-stage AD classification studies.


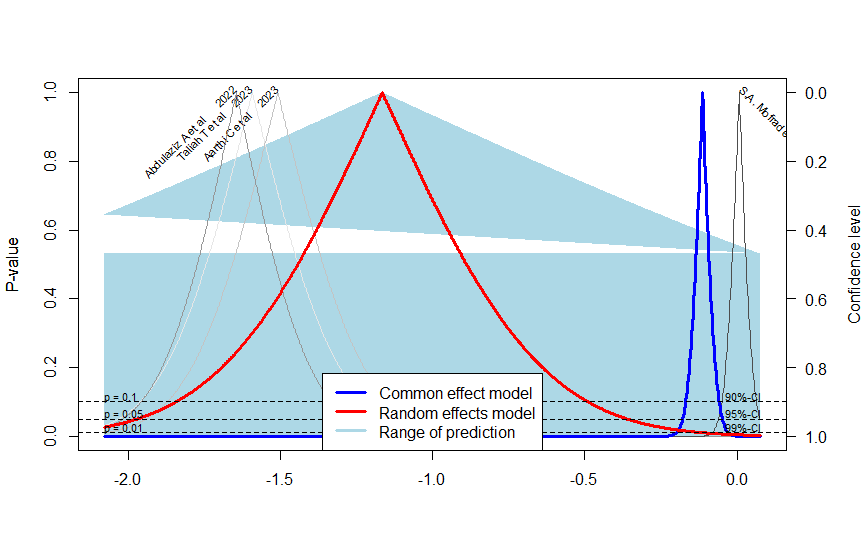


**Figure S4.** Drapery plot representation of 6-stage AD classification.
